# Climatic Niche Evolution in New World Monkeys (Platyrrhini)

**DOI:** 10.1371/journal.pone.0083684

**Published:** 2013-12-23

**Authors:** Andressa Duran, Andreas L. S. Meyer, Marcio R. Pie

**Affiliations:** 1 Laboratório de Dinâmica Evolutiva e Sistemas Complexos, Departamento de Zoologia, Universidade Federal do Paraná, Centro Politécnico, Jardim das Américas, Curitiba, Paraná, Brazil; 2 Programa de Pós-Graduação em Zoologia, Universidade Federal do Paraná, Curitiba, Paraná, Brazil; CNRS/Université Joseph-Fourier, France

## Abstract

Despite considerable interest in recent years on species distribution modeling and phylogenetic niche conservatism, little is known about the way in which climatic niches change over evolutionary time. This knowledge is of major importance to understand the mechanisms underlying limits of species distributions, as well as to infer how different lineages might be affected by anthropogenic climate change. In this study we investigate the tempo and mode climatic niche evolution in New World monkeys (Platyrrhini). Climatic conditions found throughout the distribution of 140 primate species were investigated using a principal component analysis, which indicated that mean temperature (particularly during the winter) is the most important climatic correlate of platyrrhine geographical distributions, accounting for nearly half of the interspecific variation in climatic niches. The effects of precipitation were associated with the second principal component, particularly with respect to the dry season. When models of trait evolution were fit to scores on each of the principal component axes, significant phylogenetic signal was detected for PC1 scores, but not for PC2 scores. Interestingly, although all platyrrhine families occupied comparable regions of climatic space, some aotid species such as *Aotus lemurinus*, *A. jorgehernandezi*, and *A. miconax* show highly distinctive climatic niches associated with drier conditions (high PC2 scores). This shift might have been made possible by their nocturnal habits, which could serve as an exaptation that allow them to be less constrained by humidity during the night. These results underscore the usefulness of investigating explicitly the tempo and mode of climatic niche evolution and its role in determining species distributions.

## Introduction

The advent of comprehensive databases of climatic variables [1] and the increasing availability of GIS tools has led to the proliferation of studies on predicting species geographical ranges based on their environmental tolerance [2]–[6]. These studies have been instrumental to understand how geographical distributions are delimited and how they affect overall biogeographical patterns [4], [7]–[8], particularly with respect to potentially severe anthropogenic climate changes [9]–[10]. Despite this importance, surprisingly little is known about how climatic niches change over evolutionary time [11]. For instance, much has been written about how widespread phylogenetic niche conservatism is in nature (e.g. [12]–[15]). In particular, there has been disagreement regarding whether climatic niches are conserved phylogenetically, especially in mammals (e.g. [16]–[19]). These results are of considerable importance, given that they can provide an expectation for how rapidly species can respond to recent climatic changes [17]. However, as in the case of any complex trait, climatic niches are multidimensional, and it is unlikely that all niche dimensions evolve according to the same rules. Rather, many traits have been shown to display preferred directions of change – lines of least resistance (LLR) – which strongly affect the evolutionary potential and the direction of evolution [20]. This concept has been initially proposed in the context of morphological traits such as body size [21], but has recently been extended to behavioral traits [22] and there is no reason to believe it would not apply to climatic niches. According to LLR, multidimensional traits might display preferential directions for which evolution would be facilitated if they experienced selection gradients. This bias would result from the way quantitative genetic variation is structured, such that the rate of evolution of a given trait would depend on the multivariate direction of greatest additive genetic variance within populations [15]. The existence of LLR has been largely overlooked in the literature on phylogenetic niche conservatism, and treating separately different dimensions of climatic niches might lead to a more productive discussion instead of simply testing for the presence of niche conservatism (e.g. [23]).

Primates are a suitable model system to investigate climatic niche evolution. Their relatively large body size and diurnal habits make them particularly conspicuous, such that most species are likely described and their corresponding geographical distributions are better understood than most other animal taxa [24]. The present study focuses on New World primates (infraorder Platyrrhini). This clade diverged from its sister group, the Old World primates (infraorder Catarrhini) around 47 Mya [25] and diversified across the Neotropical region from Argentina to Mexico [26]. Platyrrhini includes species with considerable variation in life history and social organization, from small pair-bonded social units of *Aotus* [27] and *Callicebus* [28] to large multi-male/multi-female of *Brachyteles* [29] and *Cacajao* [30]. These species also occupy a variety of habitats, from the dry Caatinga and Cerrados woodlands (e.g. *Callithrix jacchus*, *Callithrix penicillata*, *Cebus libidinosus*, *Alouatta caraya*) to humid rain forests (e.g. *Saguinus*, *Mico*, *Leontpithecus*) [31].

The goal of the present study is to investigate the tempo and mode of climatic niche evolution in platyrrhine monkeys. Specifically, we aim to determine the main axes of climatic niche evolution across the entire infraorder using principal component analyses and fit the resulting scores to models of trait evolution to determine the level of phylogenetic signal in each of the inferred axes.

## Materials and Methods

We obtained data from 140 New World primate species, comprising the families Callitrichidae, Atelidae, Aotidae, Pitheciidae, and Cebidae, following the taxonomy of Rylands and Mittermeier [32]. Shapefiles of species ranges were downloaded from the NatureServe database [33] and the values of 19 bioclimatic variables and one topographic variable were obtained from WorldClim GIS v 1.4 [1] at a spatial resolution of 2.5′ (∼5 km). Although being far from a complete description of all mechanisms underlying the species distributions, these bioclimatic variables should nevertheless provide a first approximation to their overall ecological tolerances (see Kamilar & Muldoon [18]). These datasets were processed in ArcGis v 9.3 [34] by generating 5000 random coordinates within the range of each species and then extracting the corresponding bioclimatic values for each point using Hawth's Analysis Tools [35]. These values were averaged and the means for all 20 variables throughout the range of each species were used in subsequent analyses. Given the high level of collinearity among bioclimatic variables, we reduced the dimensionality of the original dataset using a Principal Component Analysis (PCA) of its covariance matrix. All data were transformed into z-scores prior to the PCA to ensure that differences in measurement units (e.g. precipitation vs. temperature) would not bias the obtained results. The PCs retained for further interpretation were selected using the broken-stick method, which considers as interpretable components those with observed eigenvalues higher than the variance produced by the broken stick method [36].

The mode of evolution along each dimension of platyrrhine niche space was assessed by fitting four alternative models. In the simplest model (white noise -WN), the expected covariance among species due to their phylogenetic history is zero. Next, we used the model of Pagel [37], in which a parameter (λ) is multiplied to the off-diagonal elements of the phylogenetic variance-covariance matrix and thus measures the degree to which the trait in question evolves in a manner consistent with a simple Brownian motion model (BM). The last two tested models were designed to assess the relative fit of two specific models of trait evolution, namely stabilizing selection to a single adaptive peak, also known as an Ornstein-Uhlenbeck process (OU; [38]), and the Early Burst model (EB), in which the rate of trait evolution is accelerated during the early stages of the history of a given clade [39]. In order to identify the best-fit model, we first used Pagel's method [37] to test for phylogenetic signal in the scores of each PC using likelihood tests of BM against WN. If present, we compared the performance of BM, OU, and EB using the corrected Akaike Information Criterion (AICc), which was shown to perform well for small sample sizes [40]. These results were complemented by analyses of phylogenetic signal using Blomberg et al.'s K statistic [41]. Given that phylogenetic information is not available for all Platyrrhini, tests of alternative models of trait evolution were restricted to the 76 species available in the supertree published by Bininda-Emonds et al. [42]. All analyses were conducted using the statistical software R [43], with the packages geiger [44], phytools [45], and vegan [46].

## Results

**Figure 1:**
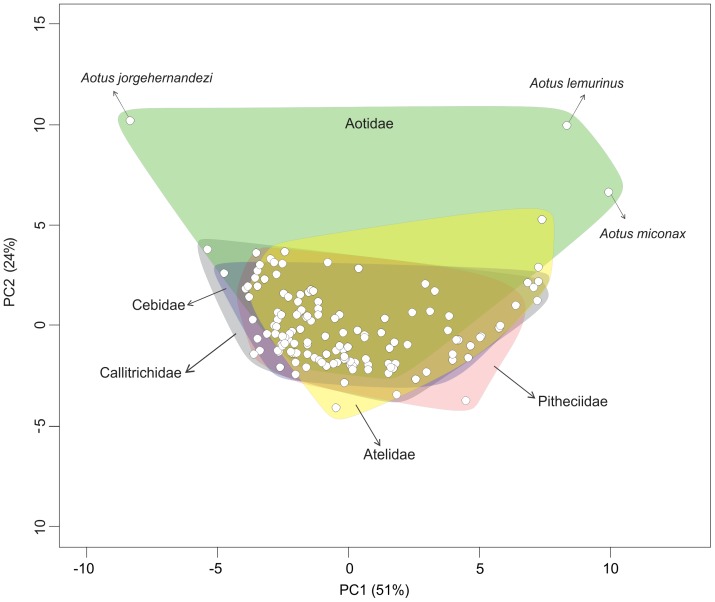
Scores along the first two PC axes representing the climatic niche space of Platyrrhini species. Each point represents the mean score for a given species.

**Figure 2:**
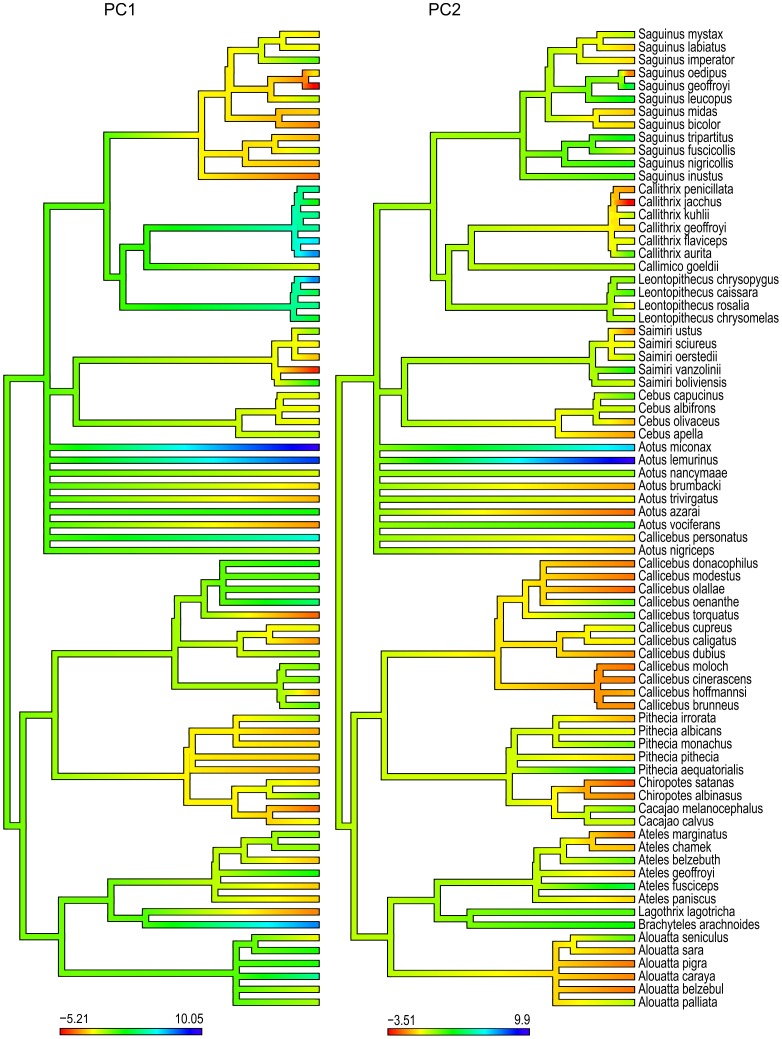
Variation in PC scores of climatic variables along the phylogeny of Platyrrhini. The first PC axis is strongly associated with temperature whereas the second PC axis is more associated with precipitation. Corresponding loadings are indicated in Table 1.

A Principal Components Analysis (PCA) was efficient in summarizing the observed interspecific climatic niches of platyrrhine monkeys, with the first two axes selected using the broken-stick method accounting for 75% of the total variance in the dataset (Table 1). Loadings on the first PC were most strongly associated with temperature, particularly during the coldest and driest months (i.e. annual mean temperature, minimum temperature of coldest month, mean temperature of driest quarter, mean temperature of coldest quarter) (Table 1). The second PC was associated with a negative relationship between levels of rainfall, particularly during the dry season (i.e. precipitation of warmest quarter, precipitation of driest quarter, precipitation of driest month) and warmer temperatures during the summer (maximum temperature of warmest month, Table 1). Interestingly, a biplot of these two PCs indicates that all platyrrhine families seem to have occupied essentially the same regions of climatic niche space, except for aotid species that score positively on PC2, such as the Colombian Night Monkey *Aotus lemurinus* (Geoffroy, 1843), the Andean Night Monkey *Aotus miconax* (Thomas, 1927), and the Hernández-Camacho's Night Monkey *Aotus jorgehernandezi* Defler & Bueno, 2007 (Figure 1). Differences in the climatic niche of platyrrhine lineages become more apparent when PC scores of each species are plotted along their phylogeny (Figure 2). In particular, differences along PC1 lead to the detection of clades characteristic of colder regions, such as *Leontopithecus* and *Callithrix*, and of warmer climates, such as *Saguinus* (Figure 2).

**Table 1:** Loadings of principal component analysis of bioclimatic variables associated with the distribution of neotropical primates.

Variable	PC1	PC2
Altitude	0.19	0.26
Annual Mean Temperature	−0.27	−0.22
Mean Diurnal Range	0.17	−0.13
Isothermality	−0.23	0.17
Temperature Seasonality	0.23	−0.04
Max Temperature of Warmest Month	−0.18	−0.35
Min Temperature of Coldest Month	−0.30	−0.08
Temperature Annual Range	0.24	−0.17
Mean Temperature of Wettest Quarter	−0.22	−0.27
Mean Temperature of Driest Quarter	−0.29	−0.16
Mean Temperature of Warmest Quarter	−0.23	−0.28
Mean Temperature of Coldest Quarter	−0.28	−0.16
Annual Precipitation	−0.25	0.21
Precipitation of Wettest Month	−0.23	0.08
Precipitation of Driest Month	−0.18	0.33
Precipitation Seasonality	0.11	−0.32
Precipitation of Wettest Quarter	−0.24	0.08
Precipitation of Driest Quarter	−0.19	0.33
Precipitation of Warmest Quarter	−0.05	0.30
Precipitation of Coldest Quarter	−0.26	0.14
Standard deviation	3.19	2.21
Proportion of Explained Variance	0.51	0.24

See text for details.

When alternative models of trait evolution were fit to each of the PCs, significant phylogenetic signal was detected for PC1 but not for PC2 scores (λ_PC1_ = 0.86, p<<0.001 and λ_PC2_ = 0.62, p<0.10, Table 2). Similar results were obtained using Blomberg et al.'s K [41] test (K_PC1_ = 0.52, p = 0.001 and K_PC2_ = 0.30 p = 0.068). Moreover, BM outperformed alternative models, showing the lowest AICc in relation to its alternatives (WN, OU, and EB; Table 2) for PC1.

**Table 2:** Fit of four macroevolutionary models with respect to platyrrhine climatic niche dimensions.

Model		PC1	PC2
White noise (WN)	logL	−196.04	−166.32
	AICc	396.25	336.80
Brownian motion (BM)	logL	−181.75	−164.98
	λ	0.86	0.62
	AICc	**369.83**	**336.29**
Ornstein-Uhlenbeck (OU)	logL	−183.72	−166.32
	α	0.07	20.22
	AICc	373.77	338.97
Early burst (EB)	logL	−186.62	−179.34
	AICc	379.59	365.02

Bold values indicate the lowest AICc estimates for each PC among the tested models. See text for details.

## Discussion

The observed variation in the climatic niches of New World monkeys showed a remarkably simple underlying structure, with average temperature (particularly during the coldest months) being the most important climatic correlate of platyrrhine geographical distributions, followed by rainfall (particularly during the dry season, Table 1), which seems to be a general phenomenon for mammals [47]. These results suggest that environmental niches might also be affected by the phenomenon of evolution along LLR [20]–[22], which would imply that evolutionary change is not equally likely in all niche axes. On the other hand, it is important to note that these differences might not necessarily mean that there are differential intrinsic physiological disparities among species with respect to changes in temperature as opposed to humidity. An alternative explanation is that the structure of the habitat itself is affected differentially by temperature in relation to humidity, and that the primates are responding more to the changes in vegetation than to the climate itself. Only an explicit comparison between how platyrrhine species and their habitat respond to variation in climate can discriminate these explanations, yet the methodological tools available for such comparison are still in their infancy [48]. However, it is important to note that these analyses are planned to detect phylogenetic niche conservatism in the realized, not the fundamental, climatic niche, which may limit the inference about ecological tolerances.

Only two studies have previously involved extensive investigations on primate climatic niche evolution. The first was conducted by Kamilar & Muldoon [18] and involved the analysis of 43 Malagasy primates. Their results differed in many respects to those observed in our study of platyrrhines, particularly: (1) niche dimensions did not separate the relative effects of precipitation and temperature in niche space; (2) no evidence of phylogenetic signal was detected in any PC. There are two main (non-exclusive) potential reasons for this discrepancy. The first is methodological: Kamilar & Muldoon [18] used individual records instead of species means to map interspecific variation in climatic niches. Although that strategy allows for more precise information on the climatic conditions where each species is found, the considerable differences among species in the number of records (6 species account for more than half of the records) might conflate intra- and interspecific variation in climatic niches. The second potential reason is biological: the age of the crown Strepsirrhini is nearly twice that of the crown Platyrrhini [25], yet Strepsirrhini evolved within a much smaller geographical area. The combination of more time for competitive interactions among lineages and opportunities for convergence within a smaller region might also have contributed to the observed difference from Platyrrhini, but the available analyses cannot discriminate among these potential sources of discrepancy. The second study involved a comprehensive analysis of many ecological and life-history traits for all primates using Blomberg et al.'s K statistic [19] and suggested consistently weak phylogenetic signal for climatic niche variables. Those results were interpreted as possibly advantageous, providing more room to evolve given possible constraints by other life-history traits with higher phylogenetic signal. Our results suggest that the low phylogenetic signal in climatic niche variables might actually reflect large-scale differences among primate lineages, such that phylogenetic signal would be stronger within different clades.

The pattern of occupation of climatic space among the studied species indicates that, in general, the entire climatic space has been thoroughly explored by all platyrrhine families, except for some species of *Aotus* (Figure 2). What biological traits in these species allowed for their occupation of such unusual region of climatic space? Further examination of the distinctive species shows that their difference from the remaining platyrrhines is concentrated on PC2, which was associated mostly with precipitation during the driest months. In particular, these *Aotus* species live in unusually dry regions, as shown by their positive scores on PC2. As indicated by their common names, *Aotus* species tend to be nocturnal and therefore are not active during the times of the day when low humidity could be a constraint. However, it is important to note that, although all *Aotus* are mostly nocturnal - some populations exhibits cathemeral activity (e.g. *A. azarai azarai*; [49]) - and only some species live in particularly dry habitats. Given that nocturnality seems to have evolved prior to the occupation of these regions of climatic space, it could represent an instance of an exaptation [50]–[51], that served as a necessary – but not sufficient – condition that allowed for the occupation of a novel region of climatic space.

The recent interest in the phenomenon of phylogenetic niche conservatism has focused disproportionately on testing whether species niches are conserved over evolutionary time (see [52]–[54]). For instance, it has been recently suggested for European mammals that climatic niches of sister species are usually not more similar to one another than expected by chance [17]. The reasons for this disagreement are difficult to ascertain, given the differences in methodology used in each study. For instance, it is possible that temperate species in particular (such as those found in Europe) have relatively broader climatic tolerances [55]. However, a common implicit assumption in many studies that did not find phylogenetic signal in climatic niches is that climatic niches respond as a single trait for which phylogenetic autocorrelation might (or might not) occur (e.g. [17], but see [56]). Our results strongly indicate that this assumption is false: phylogenetic signal seems to be considerably stronger with respect to temperature than to rainfall. In hindsight, it seems naive to assume that a complex, multidimensional trait such as the climatic niche of a species could be reduced to a single axis of variation for which significant phylogenetic signal might be present or not. Rather, we argue that heterogeneity in the tempo and mode of evolution of different axes of the climatic niche can be more prevalent than currently assumed, and understanding such heterogeneity is an important challenge for future studies on phylogenetic niche conservatism.
